# Cerebral infarction in HIV-negative patients with cryptococcal meningitis: its predictors and impact on outcomes

**DOI:** 10.1186/s12879-022-07827-z

**Published:** 2022-11-09

**Authors:** Jiashan Tu, Siyu Zhang, Qinghua Liu, Yu Lin

**Affiliations:** 1 Department of Neurology, Meizhou People’s Hospital, 63, Huangtang Road, Meijiang District, Meizhou City, 514031 Guangdong Province China; 2 Department of Radiology, Meizhou People’s Hospital, Meizhou, China

**Keywords:** Cryptococcal meningitis, HIV-negative, Cerebral infarction, Risk factors, Outcome, Magnetic resonance imaging

## Abstract

**Background:**

Descriptions of the patterns of acute/subacute cerebral infarction (ASCI) in HIV-negative patients with cryptococcal meningitis (CM) are scarce, and the predictors of ischemic stroke and outcomes following ASCI remain unclear.

**Aim:**

To study the clinical characteristics and evaluate the predictors of ASCI in HIV-negative patients with CM and assess the impact of ischemic stroke on the outcomes of the patients.

**Methods:**

We retrospectively analyzed the data of 61 HIV-negative patients with CM treated between January, 2016 and February, 2022, and among them, 53 patients with complete neuroimaging and cerebrospinal fluid (CSF) data were enrolled in this study. The cohort was stratified by the occurrence of ASCI diagnosed based on MRI evidences for comparison of the clinical characteristics (consciousness disturbance, GCS score, duration of symptoms, and treatment), CSF parameters, imaging findings (meningeal inflammation, hydrocephalus, posterior fossa exudates) and outcomes of the patients. A favorable outcome was defined as a modified Rankin scale (mRS) score ≤ 2 and a poor outcome as a mRS score > 2. Logistic regression analysis was used to identify the risk factors of ASCI in the HIV-negative patients with CM.

**Results:**

Of the 53 HIV-negative patients with CM, 14 (26.4%) had ASCI. The incidences of fever, headache, neck stiffness, duration of symptoms, CSF parameters, meningeal enhancement in brain MRI and the treatment regimens were similar between the patients with and those without ASCI. Most of the infarcts (92.9%) were of the lacunar type, involving both the anterior and posterior territories. Basal ganglia-corona radiata and the brainstem-cerebellum were the most frequently involved sites. Univariate logistic regression analysis suggested that consciousness disturbance (*P* = 0.002), MRI evidence of hydrocephalus (*P* = 0.042) and posterior fossa exudates (*P* = 0.028) were predictors of ASCI in these HIV-negative patients with CM. Multivariate analysis identified consciousness disturbance as a significant predictor of ASCI (*P* = 0.020). Compared with the patients without ASCI, the HIV-negative patients with CM and ASCI had poorer outcomes (*P* = 0.001).

**Conclusion:**

ASCI can occur in HIV-negative patients with CM, presented commonly as multiple lacunar infarctions involving all the cerebrovascular territories. The presence of consciousness disturbance, hydrocephalus and posterior fossa exudates may increase the risk of ASCI in patients with CM. ASCI is associated with a poor outcome of the HIV-negative patients with CM.

## Introduction

Cryptococcal meningitis (CM) is one of the most common fungal infections in the central nervous system (CNS), characterized by a long disease course with difficult management. Despite the advances in its diagnosis and treatment, CM is still associated with a high mortality rate (20–70%), and about 50% of the survivors have moderate to severe neurological deficits, leading to a great health care burden [1, 2]. More than 80% of CM cases occur in human immunodeficiency virus (HIV)-infected patients, but it can also be found in immunocompetent individuals [3]. In China, most of the CM cases were reported in patients without HIV infection [4].

Previous studies have shown that 13–21.5% of the patients with CM experience cerebral infarction during the course of the illness [5, 6], but for HIV-negative patients with CM, the patterns of vascular involvement, predictors of cerebral infarction and the patients’ outcomes remain poorly documented [5]. In this context, we conducted this retrospective study to evaluate the predictive factors of acute/subacute cerebral infarction (ASCI) in HIV-negative patients with CM and assess the impact of cerebral infarction on the outcomes of the patients.

## Methods

### Study design

This retrospective study was conducted among the patients diagnosed with CM identified by reviewing the hospital record of Meizhou People’s Hospital in the past 6 years (from January, 2016 to February, 2022). The Institutional Ethics Committee of the Hospital reviewed and approved the protocols of the study. Informed consent was waived by the Ethics Committee of Meizhou People’s Hospital as we used electronic medical records of the patients on an anonymous basis in this study.

### Patient enrollment and diagnostic criteria of CM

**Fig. 1:**
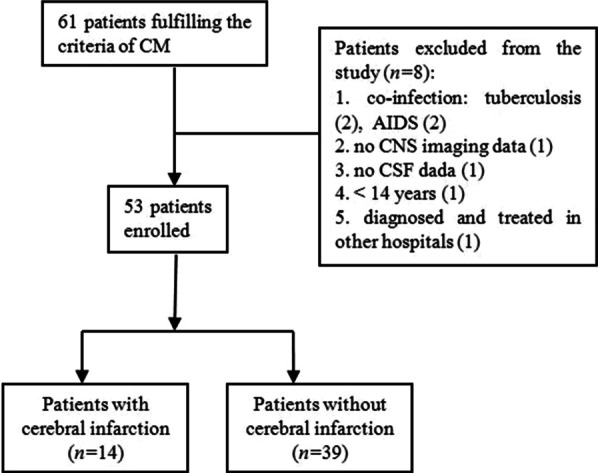
Flow chart of the patient enrollment

A total of 61 patients with CM were initially identified, among whom 53 had an early cranial magnetic resonance imaging (MRI) using a 3.0T scanner (Magnetom Skyra, Siemens Healthineers AG, Munich, Germany) at admission and were thus enrolled in this study (Fig. 1). All the enrolled patients had the first onset of CM, for which a definite diagnosis was established based on either (1) positive test results of cerebrospinal fluid (CSF), including isolation of *C. neoformans* in CSF cultures, positivity for cryptococcal antigen in the CSF, or positive India ink staining of the CSF and clinical features of meningitis; or (2) positive results of blood cultures for *C. neoformans* with clinical presentations of meningitis and typical CSF features [7].

The patients were excluded for (1) a positive test result for HIV infection; (2) an age below 14 years; (3) culture-proven co-infections (such as tuberculosis, cytomegalovirus and herpes zoster); (4) a history of antifungal therapy prior to admission; and (5) incomplete neuroimaging or CSF data.

### Treatment of CM and outcome evaluation

The patients received treatment for CM in line with the expert consensus [7] using the main antifungal regimen of liposome amphotericin B ± 5-flucytosine ± fluconazole. The induction regimen consisted of liposome amphotericin-B at a target daily dose of 0.7 mg/kg for 2 weeks combined with either 5-flucytosine at a daily dose of 100 mg/kg or fluconazole at 800 mg for 2 weeks. The patients were given oral fluconazole for consolidation therapy and maintenance treatment. The patients who could not tolerate the side effects of amphotericin B were treated with voriconazole. Extraventricular drainage and/or ventriculoperitoneal shunts were performed to relieve hydrocephalus and decrease intracranial pressure.

The endpoint of observation was the patients’ discharge from the hospital. The outcomes of the patients were assessed using a modified Rankin scale (mRS), in which a score 0 was assigned for absence of symptoms; 1 for presence of minor symptoms that did not affect daily activity; 2 for symptoms that affected lifestyle but without affecting independent living; 3 for symptoms that severely affected lifestyle and prevented independent living; 4 for symptoms that prevented independent living but full-time care was not required; 5 for total dependence on others and requirement of full-time care; and 6 for death. The patients with a score of 2 or lower were deemed to have a favorable outcome, and those with higher scores were thought to have a poor outcome.

### Demographic and clinical data collection

The demographic and clinical data of the patients were collected, including gender, age, clinical manifestations (such as fever, headache, vomiting, and neck resistance), consciousness disturbance (manifested by reduced consciousness level and changed consciousness content, such as somnolence, sopor, coma, confusion and delirium, etc.), Glasgow Coma Scale [GCS] score, seizure, cerebral hernia, and cranial nerve impairment, duration of symptoms and treatment regimens of CM. The laboratory data of CSF tests involving CSF pressure, white blood cell count, glucose, chloride and total protein were also collected.

### Diagnosis of acute/subacute cerebral infarction

New-onset cerebral infarction in the context of CM, which can either be asymptomatic or show signs of neurological deficits, was diagnosed based on brain MRI findings of the patients during hospitalization for CM treatment. The diagnostic time of ASCI was defined as the time from CM symptom onset to the confirmation of ASCI (Table 1).

**Table 1:** Characteristics and outcomes of the HIV-negative CM patients with and without acute/subacute cerebral infarctions (ASCI)

Variable	With infarct (*n* = 14)	Without infarct (*n* = 39)	*P* value
Age [years, median (range)]	49 (42, 69)	61 (41, 67)	0.610
Male:Female	8:6	28:11	0.317
Clinical features [*n* (%)]			
Fever	8 (57.1%)	24 (61.5%)	0.773
Headache	10 (71.4%)	31 (79.5%)	0.538
Vomiting	7 (50.0%)	14 (35.9%)	0.358
Consciousness disturbance	9 (64.3%)	7 (17.9%)	0.002
GCS score	14 (6, 15)	15 (15, 15)	0.015
Cerebral hernia	2 (14.3%)	0	–
Cranial nerve deficits	2 (14.3%)	4 (28.6%)	0.684
Seizures	1 (7.1%)	2 (5.1%)	0.781
Neck resistance	11 (78.6%)	20 (51.3%)	0.086
Duration of symptoms [*n* (%)]			
< 1 month	11 (78.6%)	28 (71.8%)	0.559
1–3 months	2 (14.3%)	6 (15.4%)	
> 3 months	1 (7.1%)	5 (12.8)	
ASCI diagnostic time [days, median (range)]	11 (5, 23)	–	–
CSF data [median (range)]			
Pressure (mmH_2_O)	219 (118.75, 300)	260 (170, 300)	0.439
WBC (cells/μL)	106 (44.25, 245.50)	101 (28, 184)	0.628
TP (g/L)	1.48 (0.99, 1.80)	1.07 (0.74, 1.78)	0.193
GLU (mmol/L)	1.63 (0.93, 2.55)	1.97 (1.10, 2.42)	0.716
CL (mmol/L)	113 (106.95, 117.40)	112.4 (109.0,119.2)	0.724
Treatment			
Treatment time [days, median (range)]	15.5 (5.5, 45.3)	14.0 (5, 23)	0.343
Liposome Amphotericin B [*n* (%)]	10 (71.4%)	27 (69.2%)	0.878
Fluconazole [*n* (%)]	8 (57.1%)	23 (59%)	0.905
5-Fluorocytosine [*n* (%)]	6 (42.9%)	17 (43.6%)	0.827
Voriconazole [*n* (%)]	1 (7.1%)	3 (7.7%)	0.947
MRI finding [*n* (%)]			
Meningeal inflammation	10 (71.4%)	27 (69.2%)	0.878
Hydrocephalus	7 (50%)	8 (20.5%)	0.036
Posterior fossa exudates	10 (71.4%)	14 (35.9%)	0.030
Cryptococcoma	0	1 (2.6%)	–
Poor outcome [*n* (%)]	9 (64.3%)	5 (12.8%)	0.001

*CSF* cerebrospinal fluid, *MRI* magnetic resonance imaging, *N* case number, *GCS* Glasgow Coma Scale, *CM* Cryptococcal meningitis, *WBC* white blood cell, *TP* total protein, *GLU* glucose, *CL* chloride

All the patients underwent their first cranial MRI examination during hospitalization within 1 week after admission and received a second examination if the symptoms worsened. We analyzed the main MRI features of each patient, including meningeal enhancement, posterior fossa exudates, cryptococcoma, hydrocephalus, and ASCI [6]. Meningeal enhancement was defined as the presence of leptomeningeal enhancement on contrast-enhanced T1-weighted images (T1WI) or contrast-enhanced FLAIR, and posterior fossa exudates as focal or diffuse gyriform enhancement over the basal cistern and/or the cerebellum. Hydrocephalus was defined as enlarged ventricles, dilation of the temporal horn to a size > 2 mm without obvious brain atrophy, and/or an Evan’s ratio > 0.3. Cryptococcoma was defined as mass lesions with hyperintense signals on T2WI, intermediate to low signals on T1WI, and ring or nodular enhancement on contrast-enhanced T1WI or contrast-enhanced FLAIR. ASCI was identified as lesions with hyperintensity on diffusion-weighted image (DWI) and corresponding hypointensity on apparent diffusion coefficient (ADC) maps, along with variable FLAIR hyperintensity.

### Statistical analysis

SPSS 22.0 software (SPSS Inc., Chicago, IL, USA) was used for data analysis. The Shapiro–Wilk normality test was used for testing the normality for all the quantitative variables. The data of continuous variables with a normal distribution are presented as *Mean* ± *SD* and compared using Student’s *t*-test between the two groups. The variables without normal distribution of the data are described as median with quartiles and compared using non-parametric rank sum test. The enumeration data are presented as number of cases or percentages and compared using χ^2^-test or Fisher’s exact test. Univariate logistic regression was used to evaluate the relationship between the included variables and the risk of ASCI, and multiple logistic regression was performed for the significant variables identified in univariate analysis. The variables with a zero cell count in a 2 × 2 table were eliminated from the analysis. For all analyses, a *P* value less than 0.05 was considered to indicate a statistically significant difference.

## Results

### Baseline characteristics

Table 1 shows the baseline characteristics, CSF data and neuroimaging findings of the 53 patients. Among the enrolled patients, 14 were identified to have cerebral infarction and 39 were free of cerebral infarction during the period of observation. Most of the patients were male in both of the groups. The positive rates of CSF cryptococcal antigen, India ink stain and CSF culture for *C. neoformans* in these CM patients were 86.8% (46/53), 79.2% (42/53) and 67.9% (36/53), respectively. None of the patients tested positive for HIV infection or had a history of previous stroke. As shown in Table 1, there is no statistical difference between the two groups in terms of treatment drugs or treatment time (*P* > 0.05).

### Incidence of ASCI

Neuroimaging studies identified evidence of ASCI in 14 (26.7%) of the patients. All the cerebral infarcts were acute as confirmed by MRI with DWI. Ten patients were diagnosed with ASCI within the 1st week after admission; the diagnosis of ASCI was established in another 4 patients after a second MRI examination for symptom deterioration during hospitalization. Two of the patients had mild hemiplegia and 1 patient presented with cerebellar ataxia attributable to infarction; the other 11 patients with ASCI were asymptomatic. As shown in Table 2, most of the infarcts (13/14, 92.9%) were of the lacunar type, and one patient (7.1%) had multiple large cerebral infarctions. Bilateral infarcts were seen in 8 (57.1%), unilateral infarcts in 6 (42.9%), and multiple infarcts in 10 (71.4%) of the patients. The most common site of infarction was the basal ganglia-corona radiata (57.1%, 8/14) and the brainstem–cerebellum (57.1%, 8/14), followed (in the descending order) by the frontal, thalamus, parietal, occipital, and temporal lobes. The infarcts involved both the anterior and posterior territories, mainly the brain regions supplied by the perforator arteries.

**Table 2:** Characteristics of ASCI in patients with cryptococcal meningitis

Characteristics of infarcts [n (%)]	
Single infarction	4 (28.6%)
Multiple infarction	10 (71.4%)
Unilateral infarction	6 (42.9%)
Bilateral infarction	8 (57.1%)
Lacunar infarction	13 (92.9%)
Location of infarcts	
Basal ganglia-Corona radiata	8 (57.1%)
Thalamus	3 (21.4%)
Frontal	5 (35.7%)
Parietal	2 (14.3%)
Occipital lobe	2 (14.3%)
Temporal lobe	2 (14.3%)
Brainstem	4 (28.6%)
Cerebellum	4 (28.6%)
Brainstem and cerebellum	8 (57.1%)

### Predictors of ASCI

**Fig. 2:**
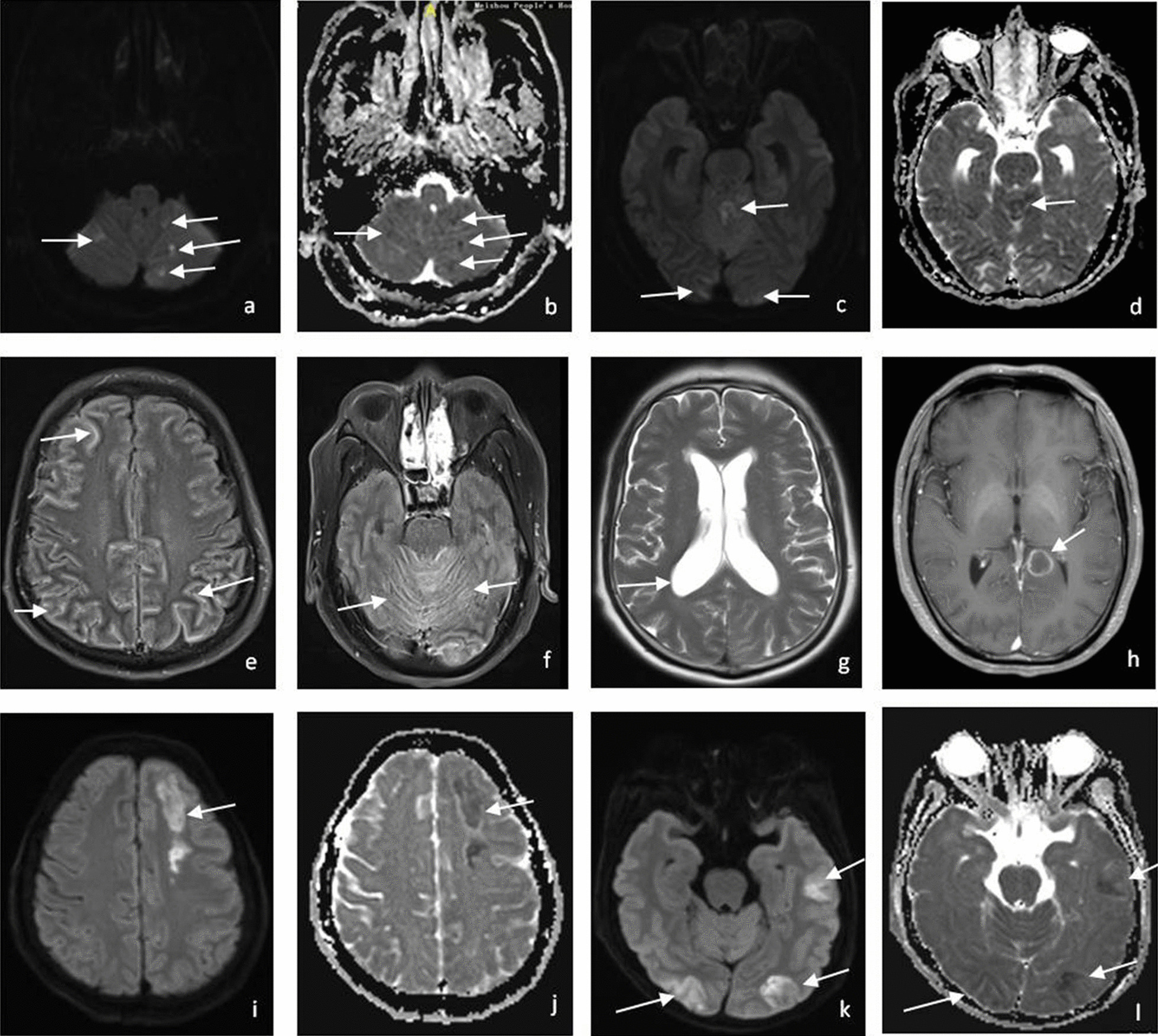
Representative MRI findings in HIV-negative patients with CM. A 64-year-old man with HIV-negative CM. **a**–**d** DWI and ADC map showing multiple lacunar infarction in cerebellar vermis, bilateral cerebellar hemispheres and bilateral occipital lobe. **e**, **f** Gadolinium (Gd)-enhanced FLAIR showing meningeal enhancement of cerebral cortex and brain stem and cerebellum (posterior fossa exudates). **g** T2WI showing ventricular dilatation (hydrocephalus). **h** Another patient with HIV-negative CM. Gadolinium (Gd) contrast enhanced T1WI showing remarkable ring enhancement in the posterior inferior region of left splenium of corpus callosum (cryptococcoma). **i**–**l** The third patient with HIV-negative CM, DWI and ADC map showing multiple large infarctions at the left frontal, left temporal, bilateral occipital lobe

In the patients with CM either with or without ASCI, the most common neuroimaging finding was meningeal enhancement, which had a similar rates between the two groups (71.4% vs 69.2%, Fig. 2). However, the incidences of posterior fossa exudates (71.4% vs 35.9%) and hydrocephalus (50% vs 20.5%) were both higher in the infarct group. Cryptococcoma was found in one patient (2.6%) without cerebral infarction and was not found in the infarct group. With regards to the clinical features, the patients with cerebral infarction had a significantly lower GCS score than those without cerebral infarction (*P* = 0.015). Univariate logistic regression analysis identified consciousness disturbance (*P* = 0.002), MRI features of hydrocephalus (*P* = 0.042) and posterior fossa exudates (*P* = 0.028) as significant predictors of ASCI in patients with CM. In multivariate analysis, only consciousness disturbance (*P* = 0.020) was found to be a significant factor associated with the presence of ASCI (Table 3).

**Table 3:** Univariate logistic analysis of the potential risk factors for acute/subacute cerebral infarction in patients with cryptococcal meningitis

Variable	OR	95% CI	*P* value
GCS score	0.876	0.760– 1.008	0.065
Consciousness disturbance*	8.229	2.101–32.229	0.002*
Hydrocephalus	3.875	1.051–14.282	0.042
Posterior fossa exudates	3.929	1.029–14.992	0.045

^*^P = 0.020 on multivariate analysis

### Outcomes of the patients

In the 14 patients with ASCI, 9 showed deterioration of CM symptoms and had poor outcome. Four (28.6%) of the patients were discharged with endotracheal intubation eventually, and 5 (35.7%) were bedridden requiring full-time care. In the patients without ASCI, 3 were discharged with endotracheal intubation. Table 1 shows that compared with those without ASCI, the patients with ASCI had a significantly higher percentage of poor outcomes (64.3% vs 12.8%, *P* = 0.001). The odds ratio of poor outcome was 12.2 for patients with neurovascular involvement over those without ASCI.

## Discussion

ASCI can occur in patients with CM, but its presentations are often masked by the manifestations of CM [5, 6, 8]. Mishra et al. [5] reported that the incidence of cerebral infarction was 13% in CM patients, including both HIV-positive and HIV-negative patients, but only 30% of the patients in their study underwent brain MRI, and the routine CT examination has a relatively low detection rate for small acute infarcts. In the study conducted by Chen et al. [8] 37 out of 54 adult HIV-negative patients with CM had brain MRI at admission, and the detection rate of ASCI was 18.9%. Wan-Chen et al. [6] reported an ASCI incidence of 21.5% in adult HIV-negative patients with CM based on brain MRI findings using a 1.5T scanner. In this study, we recorded a higher ASCI incidence of 26.4% among the 53 patients, possibly because all the patients underwent brain MRI studies using a 3.0T scanner early after admission, and the high field MRI with a high resolution is more sensitive for detecting small infarcts.

Most of our patients with ASCI had infarctions of the lacunar type (13/14, 92.9%); multiple cerebral infarctions occurred in the majority of the patients with ASCI (10/14, 71.4%), and over half (8/14, 57.1%) of the patients had bilateral infarcts. These findings suggest that in CM patients, cerebral infarction occurred most likely in the brain region supplied by a perforator-type artery, which may explain the relatively high frequency of multiple lacunar infarctions in these patients. We noted a discrepancy concerning the infarct type from a previous report, where all the infarcts were of the lacunar type and 70% of them were unilateral [5]. This discrepancy may partly be attributed to a high rate (65%) of HIV infection in the enrolled cases in the reported study, as neurovascular complications may occur in CM patients with HIV infection through different mechanisms from those without HIV infection [5]. We found multiple large cerebral infarction only in one patient (7.1%), a rate much lower than those in previous studies [5, 8, 9]. Although lacunar infarction is the most common type of ASCI in HIV-negative patients with CM, cases of large infarctions due to large vessel involvement had been reported, as a result of direct vascular invasion or secondary to associated inflammations [10, 11].

Our finding suggests that in CM patients, the most common site of infarction was the basal ganglia-corona radiate (57.2%, 8/14), followed by the frontal lobe, brainstem, cerebellum, thalamus, parietal lobe, occipital lobe, and the temporal regions. We observed a relatively high incidence of brainstem-cerebellum involvement in CM patients with ASCI (57.2%, 8/14), and this posterior circulation infarction used to be thought uncommon in CM [12]. A previous study suggests that the brain regions supplied by the middle cerebral artery are the most frequent regions of infarct occurrence, and the vertebrobasilar system is less frequently affected by bathing in the basal exudates [5]; but our finding indicates otherwise: infarction can occur in both the anterior and posterior cerebrovascular territories in HIV-negative patients with CM. This difference may be due to our high detection rates (10/14, 71.4%) of posterior fossa exudates, which increase the risk of infarction at the corresponding position. Kumar et al. [13] suggested that caudate head and cerebellar infarctions were also common in CM, which was consistent with our finding.

Posterior fossa exudates was one of the main cranial MRI findings in our patients and a significant factor contributing to the development of ASCI. Wan-Chen Tsai et al. [6] concluded that basal meningeal enhancement was associated with the occurrence of ASCI, possibly due to the intense inflammatory reactions in the adjacent small vessels elicited by meningeal exudates, especially in the basal and cerebellar meninges. Zimelewicz et al. [14] reported a case of central nervous system vasculitis in an immunocompetent CM patient, who showed exudate in the posterior cranial fossa on MRI and bilateral atypical vasculitis in the posterior cranial fossa by digital angiography. In this study, the high detection rate of posterior fossa exudates among the patients with ASCI provides further evidence to support the presumption that posterior fossa exudates increases the risk of ASCI in HIV-negative CM patients.

We found that hydrocephalus was independently associated with the occurrence of ASCI in HIV-negative CM patients. According to the study of Chen et al. [9], hydrocephalus might occur in CM patients (due to the inflammatory exudates, fungal polysaccharides and high protein levels) to cause CSF accumulation in the arachnoid villi or subarachnoid space to block CSF outflow and absorption. Hydrocephalus may stretch the local compromised arteries and potentially causes cerebral infarction [9]. Our finding provides further evidence that hydrocephalus increases the risk of ASCI in HIV-negative CM patients.

Here we show that the CM patients with ASCI had significantly lower GCS scores than those without ASCI (*P* = 0.015). As GCS scores can not fully evaluate consciousness status of the patients before coma onset, we used the parameter consciousness disturbance for multivariate logistic analysis. Table 3 shows that consciousness disturbance was a significant risk factor for ASCI in the CM patients (*P* = 0.02). Previous studies also demonstrated that CM patients with ASCI had a higher frequency of consciousness disturbance and a higher rate of positive CSF culture for cryptococcus [2, 8]. Kumar et al. [13] proposed that altered consciousness on admission was associated with an increased risk of infarction. We presume that consciousness disturbance increases the disease burden and causes more intense vascular inflammation to contribute to cerebral infarction in CM patients.

Currently the correlation between CM and cerebral vasculopathy remains unclear. Several mechanisms have been proposed to explain the association between CM and cerebral infarctions. First of all, inflammatory changes in the vascular endothelium, resulting directly from the toxic effect of the *Cryptococcus* and from vasculitis due to antigen–antibody complex deposition, are most likely to cause vascular complications in CM. Exudative meningitis in the posterior fossa results in strangulation, vasospasm, constriction, periarteritis and even necrotizing panarteritis of the blood vessels, leading eventually to thrombosis and occlusion [14–16]. Secondly, the extensive inflammatory fibrosis of the subarachnoid space caused by *Cryptococcus* may mechanically compress the small veins, and thus increases blood flow resistance [17]. Thirdly, the inflamed vessels are stretched due to dilation of the ventricles [18], as shown by a higher incidence of hydrocephalus in the patients with ASCI than those without ASCI (50% vs 20.5%) in this study. In addition, cryptococcal infections are also thought to precipitate the vulnerability of atherosclerotic plaques and hence lead to infarction [19]. Finally, multiple acute infarctions are very likely secondary to septic embolism or mechanical obstruction [20, 21]. These assumptions provide possible explanations for the wide distribution of ASCI across both the anterior and posterior cerebrovascular territories in our CM patients, but the exact mechanisms of ASCI in these patients still remain to be further investigated.

In this study, 9 (64.3%) of the 14 patients with ASCI had poor outcomes. Cerebral infarction, along with other factors such as cerebral hernia, consciousness disorder, hydrocephalus, visual impairment, and increased intracranial pressure, all independently determines the poor outcomes of CM [21–23]. Chen et al. [8] reported poor outcomes of 7 CM patients with ASCI, among whom 3 died, 2 were bedridden, and 2 were wheelchair bound. The high rate of poor outcomes in that report may result largely from the failure of early detection of asymptomatic ASCI because not all the patients enrolled had MRI examination. We also found that the CM patients with ASCI had a significantly higher likelihood of having poor outcomes than those without ASCI (64.3% vs 12.8%), which is consistent with the findings of previous studies [21–24].

## Conclusions

In summary, cerebral infarction in HIV-negative patients with CM often presents as multiple lacunar infarctions involving all the cerebrovascular territories. Conscious disturbance, hydrocephalus, and posterior fossa exudates are all significant predictors of ASCI in CM patients, who often have unfavorable outcomes. But in view of the retrospective nature and the relative small sample size of this study, the reliability of our conclusions remains to be further tested by future studies in more balanced cohorts and larger sample sizes.

## Data Availability

The data used to support the findings of this study are available from the corresponding author upon request.

## References

[1] Rajasingham R, Smith RM, Park BJ (2017). Global burden of disease of HIV-associated cryptococcal meningitis: an updated analysis. Lancet Infect Dis.

[2] Zhong Y, Zhou Z, Fang X (2017). Magnetic resonance imaging study of cryptococcal neuroradiological lesions in HIV-negative cryptococcal meningitis. Eur J Clin Microbiol Infect Dis.

[3] Williamson PR, Jarvis JN, Panackal AA (2017). Cryptococcal meningitis: epidemiology, immunology, diagnosis and therapy. Nat Rev Neurol.

[4] Wei F, Fa Z, Liao W (2015). Epidemiology of Cryptococcus and cryptococcosis in China. Fungal Genet Biol.

[5] Mishra AK, Arvind VH, Muliyil D (2018). Cerebrovascular injury in cryptococcal meningitis. Int J Stroke.

[6] Wan-Chen T, Chia-Yi L, Jun-Jun L (2018). The prognostic factors of HIV-negative adult cryptococcal meningitis with a focus on cranial MRI-based neuroimaging findings. J Clin Neurosci.

[7] Liu ZY, Wang GQ, Zhu LP (2018). Expert consensus on the diagnosis and treatment of cryptococcal meningitis. Zhonghua Nei Ke Za Zhi.

[8] Chen SF, Lu CH, Lui CC (2011). Acute/subacute cerebral infarction in HIV-negative adults with cryptococcal meningoencephalitis (CM): a MRI-based follow-up study and a clinical comparison to HIV-negative CM adults without ASCI. BMC Neurol.

[9] Chen YF, Wang DN, Chen ZT (2016). Risk factors associated with acute/subacute cerebral infarction in HIV-negative patients with cryptococcal meningitis. J Neurol Sci.

[10] Cachia D, Singh C, Tetzlaff MT (2015). Middle cerebral artery territory infarct due to cryptococcus infectionstitle: an uncommon indication for cerebrospinal fluid analysis in stroke patients. Diagn Cytopathol.

[11] Tahhan F, Haritounian A, Duong L (2021). Cryptococcus meningoencephalitis with severe localizing neurological deficits mimicking a large right middle cerebral arteryinfarct in a HIV patient. Cureus.

[12] Mishra AK, Vanjare HA, Raj PM (2017). Cryptococcal meningitis presenting as acute onset bilateral cerebellar infarct. J Neurosci Rural Pract.

[13] Kumar M, Dhar N, Tiwari A (2021). Comparison of patterns of infarction in TB and cryptococcal meningitis. Trans R Soc Trop Med Hyg.

[14] Zimelewicz Oberman D, Patrucco L, Cuello OC (2018). Central nervous system vasculitis for Cryptococcosis in an immunocompetent patient. Diseases.

[15] Shi M, Li SS, Zheng C (2010). Real-time imaging of trapping and ureasedependent transmigration of Cryptococcus neoformans in mouse brain. J Clin Invest.

[16] Sood V, Pattanashetti N, Ramachandran R (2019). Deceptively asymptomatic cryptococcaemia in a renal transplant recipient: the lull before a storm. BMJ Case Rep.

[17] Shimoda Y, Ohtomo S, Arai H (2017). Subarachnoid small vein occlusion due to inflammatory fibrosis-a possible mechanism for cerebellar infarction in cryptococcal meningoencephalitis: a case report. BMC Neurol.

[18] Liliang P-C, Liang C-L, Chang W-N (2003). Shunt surgery for hydrocephalus complicating cryptococcal meningitis in human immunodeficiency virus-negative patients. Clin Infect Dis.

[19] Kang A, Haynor D (2015). MR angiography of large-vessel intracranial stenosis after cryptococcal meningitis. Radiol Case Rep.

[20] Peng TJ, Kimbrough T, Tolchin BD (2019). Clinical Reasoning: a 71-year-old man receiving treatment for cryptococcal meningitis, developing new-onset lethargy. Neurology.

[21] Polk C, Meredith J, Kuprenas A (2020). Cryptococcus meningitis mimicking cerebral septic emboli, a case report series demonstrating injection drug use as a risk factor for development of disseminated disease. BMC Infect Dis.

[22] Yang H, Yin F, Xiao T (2020). A correlation analysis between clinical manifestations, therapeutic strategies, and the prognosis of children with cryptococcal meningitis in China. Int J Infect Dis.

[23] Wu L, Xiao J, Song Y (2020). The clinical characteristics and outcome of cryptococcal meningitis with AIDS in a tertiary hospital in China: an observational cohort study. BMC Infect Dis.

[24] Qu J, Zhou T, Zhong C, Deng R (2017). Comparison of clinical features and prognostic factors in HIV-negative adults with cryptococcal meningitis and tuberculous meningitis: a retrospective study. BMC Infect Dis.

